# The Story of the Insane from Year to Year

**Published:** 1904-04-30

**Authors:** 


### THE STORY OF THE INSANE FROM YEAR TO YEAR.

Sixteenth Annual Series.

### LEBANON HOSPITAL FOR THE INSANE AT BEYROUT.

At the beginning of the year this hospital contained 32 patients, and at the end of the year 39 were in residence. There were 11 recoveries, 15 were improved, and 8 died. Most of the patients were admitted from Beyrout or from Mount Lebanon, but 6 were from Damascus, and solitary cases were sent in from Jerusalem, Alexandria, Jaffa, Aleppo, etc. As regards religion, 23 were of the Greek Church, 2 were Protestants, 8 were Catholics, 19 were Maronites, 7 were Moslems, 2 were Armenians. Jews and Metwalys were also represented, and it would seem as if Dr. Wolff had as heterogeneous a collection of patients as ever fell to the lot of any medical superintendent. The Americans have shown much interest in the welfare of this hospital. Funds have been subscribed in the United States for the erection of a new block for acute female cases, and this will probably have been opened before this notice is in print. Dr. Bawn, an American lady doctor, bore the expense of putting up a similar block for acute male cases, and this was opened during last year. Switzerland sent sufficient money to buy an additional piece of land, but another £250 is wanted to make the site up to 60 acres in extent, and it should certainly not be less. We are pleased to be able to chronicle that England has not been backward in helping on the good work, and last year sent upwards of £1,000. Many of the patients admitted are extremely poor and are unable to contribute towards their maintenance, and the committee most truly say that what is wanted to free the hospital from financial strain is an endowment fund, and the requisite sum is named at £30,000. Donations or subscriptions will be received by the treasurer, Sir Richard Tangye, or by the secretary, Mr. F. C. Brading, at 35 Queen Victoria Street, E.C.

### WEST RIDING ASYLUM AT WADSLEY, NEAR SHEFFIELD.

On the first of January there were in residence here 1,670 patients, and at the end of 1902 this number had increased by 3 only. There were 425 first admissions, 106 readmissions, 192 recoveries, 66 discharged relieved, 71 discharged not improved, and 199 died. The total number under treatment was 2,192, and the average number resident was 1,659. The recoveries work out at 36·1 per cent. of the admissions, and the deaths at 11·9 per cent. of the average number resident. Of the year's deaths, 108 were caused by cerebral and spinal diseases, 12 by pneumonia, 30 by consumption, and 5 by colitis. Of the admissions, 221 were supposed to owe their insanity to various moral causes, chiefly domestic trouble, adverse circumstances, and mental anxiety. Intemperance in drink with 88, and hereditary influence with 132, were among the most potent of the physical-causes. Dr. Kay says that phthisis "largely contributed to the death-rate and that this strongly points to the necessity of making some suitable and separate accommodation for these cases." The bacterial treatment of sewage has been tried at this asylum, but has not been found as successful as was anticipated, and the drains are now connected with the Worthley district sewers. Two attendants and four nurses received pensions during the year, but the amounts given are not stated. The committee "are pleased to report that Dr. Kay and the other medical officers, together with the official staff, have given every care to the patients." The weekly cost per head is 9s. 8d.

### LONDON COUNTY ASYLUM AT COLNEY HATCH.

On the first of January this asylum contained 2,500 patients, and 2,487 were under treatment at the end of the year. There were 391 first admissions, 86 readmissions, 178 recoveries, 74 discharged relieved, and 238 deaths. The total number under treatment during the year was 2,977, and the average daily number resident was 2,490. The recoveries, excluding transfers, were 40 per cent. of the admissions, and the deaths were 9·55 per cent. of the average number resident.

Of the year's deaths 68 were caused by cerebral and spinal diseases, 38 by phthisis, 19 by pneumonia, 8 by other lung diseases, and 25 by colitis. Of the year's admissions the insanity was supposed to be caused by moral causes in 70 instances, and among the physical causes drink accounted for 63 and hereditary influence for 111. Smallpox broke out, but was fortunately limited to three cases, all of whom recovered. There were five cases of enteric fever, two being patients and three being nurses. Of the latter, one died. There were 65 cases of colitis, all being patients; and it is curious to note how often this disease limits itself to the insane and passes the staff, while enteric fever shows no such preference. Dr. Seward says that no sanitary defect could be discovered which could account for the enteric. A ward has been set apart for the treatment of the female consumptive cases. The committee say they "have much pleasure in again testifying to the able manner in which the asylnm has been administered by the medical superintendent, assisted by the medical and general staff." The weekly cost of maintenance was a little over 11s. 8d. per head.

